# Effects of nutrition therapy on growth, inflammation and metabolism in immature infants: a study protocol of a double-blind randomized controlled trial (ImNuT)

**DOI:** 10.1186/s12887-020-02425-x

**Published:** 2021-01-07

**Authors:** Kristina Wendel, Helle Cecilie Viekilde Pfeiffer, Drude Merete Fugelseth, Eirik Nestaas, Magnus Domellöf, Bjorn Steen Skålhegg, Katja Benedikte Presto Elgstøen, Helge Rootwelt, Rolf Dagfinn Pettersen, Are Hugo Pripp, Tom Stiris, Sissel J. Moltu, the ImNuT Collaboration Group, Marlen Fossan Aas, Mona Kristiansen Beyer, Jens-Petter Berg, Marianne Bratlie, Atle Bjornerud, Maninder Singh Chawla, Siw Helen Westby Eger, Cathrine Nygaard Espeland, Oliver Geier, Gunnthorunn Gunnarsdottir, Christina Henriksen, Per Kristian Hol, Henrik Holmstrøm, Ivan Maximov, Tone Nordvik, Madelaine Eloranta Rossholt, Helene Caroline Dale Osterholt, Ingjerd Saeves, Elin Blakstad, Henriette Astrup, Helge Froisland, Lars Tveiten, Krzysztof Hochnowski, Terje Reidar Selberg, Henning Hoyte, Randi Borghild Stornes, Hanne Isdal, Thea Wauters Thyness, Petra Huppi, Alexandre Lapillonne

**Affiliations:** 1 Department of Neonatal Intensive Care, Oslo University Hospital, Oslo, Norway; 2 Department of Pediatric Neurology, Oslo University Hospital, Oslo, Norway; 3 Institute of Clinical Medicine, University of Oslo, Oslo, Norway; 4 Department of Pediatrics, Vestfold Hospital Trust, Tønsberg, Norway; 5 Department of Clinical Sciences, Pediatrics, Umea University, Umea, Sweden; 6 Division of Molecular Nutrition, Department of Nutrition, University of Oslo, Oslo, Norway; 7 Department of Medical Biochemistry, Oslo University Hospital, Oslo, Norway; 8 Norwegian National Unit for Newborn Screening, Division of Pediatric and Adolescent Medicine, Oslo University Hospital, Oslo, Norway; 9 Oslo Centre of Biostatistics and Epidemiology, Oslo University Hospital, Oslo, Norway

**Keywords:** Arachidonic acid, Docosahexaenoic acid, Preterm, Nutrition, Brain maturation, Inflammation

## Abstract

**Background:**

Current nutritional management of infants born very preterm results in significant deficiency of the essential fatty acids (FAs) arachidonic acid (ARA) and docosahexaenoic acid (DHA). The impact of this deficit on brain maturation and inflammation mediated neonatal morbidities are unknown. The aim of this study is to determine whether early supply of ARA and DHA improves brain maturation and neonatal outcomes in infants born before 29 weeks of gestation.

**Methods:**

Infants born at Oslo University Hospital are eligible to participate in this double-blind randomized controlled trial. Study participants are randomized to receive an enteral FA supplement of either 0.4 ml/kg MCT-oil™ (medium chain triglycerides) or 0.4 ml/kg Formulaid™ (100 mg/kg of ARA and 50 mg/kg of DHA). The FA supplement is given from the second day of life to 36 weeks’ postmenstrual age (PMA). The primary outcome is brain maturation assessed by Magnetic Resonance Imaging (MRI) at term equivalent age. Secondary outcomes include quality of growth, incidence of neonatal morbidities, cardiovascular health and neuro-development. Target sample size is 120 infants (60 per group), this will provide 80% power to detect a 0.04 difference in mean diffusivity (MD, mm^2^/sec) in major white matter tracts on MRI.

**Discussion:**

Supplementation of ARA and DHA has the potential to improve brain maturation and reduce inflammation related diseases. This study is expected to provide valuable information for future nutritional guidelines for preterm infants.

**Trial registration:**

Clinicaltrials.gov ID: NCT03555019. Registered 4 October 2018- Retrospectively registered.

## Background

Preterm birth is the leading cause of child mortality in high and middle-income countries [1]. Very preterm infants need a combination of enteral and parenteral nutrition to meet their nutritional requirements during hospitalization. Replacing the nutrition provided by the placenta has proven difficult, resulting in postnatal growth restriction [2]. Growth and maturation of organs during the last trimester rely on a steady supply of nutrients. Inadequate supply may lead to neurodevelopmental impairment, chronic lung disease, altered host defense, hypertension, and insulin resistance [3, 4]. The main target for feeding preterm infants is to achieve growth resembling normal fetal growth rates [5] as well as satisfactory functional development [6]. Despite established international recommendation, the nutritional management varies considerably between countries, hospitals, and even within institutions [7, 8]. Training in use of parenteral nutrition (PN) and standardization of nutritional management is important to improve the implementation of nutritional guidelines [8]. Improving the quality and quantity of nutrition provided to extreme premature infants during their critical period of somatic growth and metabolic programming may be pivotal for short-term clinical outcomes as well as long-term neurodevelopmental, cardiovascular and metabolic health. In 2010 a randomized, controlled trial conducted in our institution (the PRENU study) investigated the effect of enhanced nutrient supply, including arachidonic acid (ARA) and docosahexaenoic acid (DHA), in very low birth weight (VLBW) infants compared to standard diet. The intervention group showed significant higher in-hospital growth rates and catch-up growth in head circumference (HC) from birth to 36 weeks PMA [9] as well as improved brain maturation on Magnetic Resonance Imaging (MRI) at term equivalent age (TEA) [10]. Of note, this study was discontinued early due to a high occurrence of a refeeding like syndrome among the intervention infants [11]. The risk of refeeding like syndrome has been confirmed by others [12–15] and the early need for phosphate and potassium supplementation is highlighted in the revised European guidelines on Pediatric Parenteral Nutrition [16, 17]. Moreover, this underlines the importance of conducting well-designed trials on nutritional management in this patient population.

The long chain polyunsaturated fatty acids (LC-PUFAs) linoleic acid and α-linolenic acid are essential fatty acids (FAs) that must be supplied through the diet [18]. They provide energy and are used as precursors of the LC-PUFAs; ARA, DHA and eicosapentaenoic acid (EPA). Particularly ARA and DHA accumulate in the brain during the last trimester and the first postnatal months, i.e. the period of rapid growth and brain development [19]. DHA is one of the main building blocks of the central nervous system including retina and comprises 30–50% of neuronal plasma membranes by weight [20]. Extremely premature infants have low endogenous capacity for conversion of linoleic acid and α-linolenic acid to ARA, DHA and EPA [21]. The lack of adipose stores and limited provision of essential fatty acids through the parenteral solutions increase the risk of depletion. DHA deficiency may lead to reduced visual function and alterations in behavior or cognitive performance [22]. DHA and ARA supplementation in very preterm infants have shown positive effects on growth, visual function and mental development [23].

LC-PUFAs are not only essential cellular building blocks and important sources of energy, but they also act as signal molecules, important in sustaining and resolving inflammation [24]. Studies show that immature infants have elevated levels of inflammatory cytokines during the neonatal period, and that upregulated cytokine expression is associated with the development of bronchopulmonary dysplasia (BPD), patent ductus arteriosus (PDA), retinopathy of prematurity (ROP), necrotizing enterocolitis (NEC), white matter injury (WMI) of the brain and impaired neurodevelopmental outcomes [25–29]. A proposed mechanism behind this up-regulated immune response is sustained activation and impaired resolution of inflammation [27]. There is growing evidence that in addition to structural effects on growth and organ development, supplementation with ARA and DHA, may reduce the incidence or severity of BPD, ROP, NEC and WMI by modulating the immune response [30–32]. Both omega-6 (ARA) and omega-3 LC-PUFAs (DHA, EPA) serve as precursors for the synthesis of bioactive mediators involved in immune modulation. ARA is a precursor of pro-inflammatory mediators (such as leukotrienes of the n-4 series), and of prostaglandins and thromboxanes of the n-2 series, which increase the vascular tone and promote platelet aggregation. However, ARA is also a precursor of lipoxins which are inflammation resolving mediators. Metabolites from DHA and EPA can modulate inflammation by decreasing the production of pro-inflammatory cytokines (TNF-α, IL-1β and IL-6) through the peroxisome proliferator-activated receptor (PPAR) pathways. This in turn inhibits the nuclear transcription factor κB (NF-κB) and increases the production and secretion of anti-inflammatory eicosanoids such as interleukin-10 [32]. Resolvins, protectins, and maresins formed from both DHA and EPA evoke anti-inflammatory and pro-resolving mechanisms, and they enhance microbial clearance [31].

Perinatal infections or inflammation processes play an important role in the pathogenesis of several comorbidities associated with preterm birth, such as BPD, PDA, ROP, NEC and WMI [33]. Very preterm infants are susceptible to septicemia, possibly as a result of attenuated innate immune responses [27]. Interestingly, these infants also show signs of sustained systemic inflammation with elevated pro-inflammatory cytokines [25–27, 34]. Septicemia may be defined as “the host’s deleterious and non-resolving systemic inflammatory response to microbial infection” [35]. The host response is similar to the activation triggered by non-infectious tissue injuries like surgery and ischemic reperfusion events [36]. The alarmin molecule, High Mobility Group Box 1 (HMGB1), is an activator of NF-κB and has been recognized as an important mediator of sepsis [36] and lung injury in preterm infants [37]. HMGB1 is released by necrotic cells, and sustains the inflammatory process after the resolution of the early stage of inflammation [37]. As mentioned, one of the anti-inflammatory potentials of Omega 3-PUFAs is the ability to inhibit the activation of NF-κB [32], and thereby possibly modulate an inappropriate inflammatory response.

The pathogenesis of BPD is multifactorial, but intrauterine and postnatal growth restriction is an independent risk factor [38] disturbing pulmonary alveolar and vessel growth [39]. Along with sufficient early supply of protein and energy to promote growth, omega-3 PUFAs seem to protect against lung injury or reduce BPD severity by a DHA dependent activation of the PPAR pathways [37, 40], thereby accelerating lung maturation, pneumocyte growth and vasoproliferation [40]. Studies show conflicting results. Some studies suggest that low DHA blood levels in premature infants are associated with increased incidence of BPD [41] and that fish oil supplementation may improve lung function [42, 43]. However, one study with enteral supplementation with 60 mg/kg/d of DHA did not result in a lower risk of BPD among preterm infants as compared to standard DHA intake and may have even resulted in a greater risk [44]. A controversy is the importance of balancing the amounts of ARA and DHA, since DHA supplementation alone may suppress ARA concentrations. Fetal plasma levels of ARA are high, with an ARA:DHA ratio around 3:1 at the beginning of the 3rd trimester compared to about 2:1 in term infants. A low ARA:DHA ratio in extreme preterm infants (GA < 28 weeks) has been associated with more severe BPD [45].

ROP is a disorder of vascular development of the retina and the main reason for visual impairment in extreme premature infants. As for the lung, both nutritional and inflammatory factors seem to be important mediators in disease progression. DHA is a major structural lipid in retina and accounts for approximately 50–60% of the total fatty acid content within rod outer segments of photoreceptors [46]. Small RCTs have shown that early lipid supply reduces the incidence of ROP in VLBW infants [47, 48]. Two studies have demonstrated a significantly reduced incidence of ROP with fish-oil containing lipid emulsion as compared to standard soybean oil or a soybean and olive oil emulsion [49, 50]. On the contrary, a trial that compared a multicomponent lipid emulsion (soybean oil, olive oil, fish oil and middle chain triglycerides) with a soybean and olive oil emulsion on the prevalence of ROP in extremely premature infants did not show any differences between the groups [51]. Both decreased levels of DHA and ARA were associated with the development of ROP. A recent RCT showed that enteral supplementation with DHA significantly reduced the incidence of stage 3 ROP in premature infants [52].

WMI of the brain accounts for the predominance of neurological sequelae in surviving premature infants, including cerebral palsy and cognitive deficits [53]. The two main mechanisms presumably responsible for the degeneration of immature oligodendrocytes are hypoxia-ischemia and inflammation [54]. WMI of the premature brain include axonal damage, necrosis and periventricular leukomalacia (PVL) and is commonly categorized in diffuse WMI and focal WMI. MRI defined diffuse WMI is poorly understood histopathological, but is thought to mainly result from damaged oligodendrocytes and less from axonal damage [54]. In both forms of WMI an activation of microglia and astrocytes, as a diffuse inflammatory response is common [55]. Clinically, WMI is associated with hemodynamic instability, poor postnatal growth, and inflammation [34, 54], suggesting that measures to optimize nutrition and reduce inflammation might be beneficial in disease prevention. Other common neurologic comorbidities in the preterm infant includes germinal matrix hemorrhages, intraventricular hemorrhages (IVH) and diffuse atrophy, the cause of which are multifactorial. Interestingly, inflammatory microglial and astrocytic activation following IVH has also been shown to be a determinant of white matter brain damage in preterm infants [56], and early increased cytokine levels in serum is associated with the development of IVH [57]. Hence, we hypothesize that supplying the essential fatty acids ARA and DHA will improve both brain maturation on MRI at TEA as well as overall brain MRI morbidity score ad modum Kidokoro [58].

NEC is a serious disease of the intestines in very preterm infants and may lead to intestinal failure or death. As in the above mentioned comorbidities, the pathogenesis is multifactorial and numerous inflammatory mediators seem to play a prominent role [28, 59]. A few studies show reduced incidence of NEC with enhanced early lipid supply to VLBW infants [47, 48], and a systematic review of omega-3 PUFAs for extremely preterm infants found a trend toward a reduction in the risk of NEC [22].

Increasing evidence indicates that preterm birth and intrauterine growth restriction (IUGR) affects endocrine and metabolic adaptation that program cardiovascular diseases and type 2 diabetes in adult life, the smallest neonates having the highest risk [60, 61]. The embryonic and fetal heart development mainly involves the proliferation of mononucleated cardiomyocytes. The proliferative capacity is lost shortly after birth and the continuing heart growth is due to an increase in cardiomyocyte volume. Thus, by the early neonatal period the human heart contains almost the full complement of cardiomyocytes for the rest of life [62]. Altered myocardial structures have been found in association with intrauterine growth restriction and preterm birth [63]. The limited capacity for cellular regeneration within the postnatal heart after injury may have long-term consequences for heart development [64].

Further studies on the optimal fatty acid composition for nutritional therapy in extremely preterm infants are warranted. Based on this, we designed a double-blind RCT to determine whether early and prolonged supply of ARA and DHA improves brain maturation, quality of growth and clinical outcomes in extreme premature infants as compared to our present nutrient supply.

## Methods/design

### Study design

A single center, parallel group, double-blind randomized controlled trial. The intervention group receives a lipid supplement containing DHA and ARA, and the control group receives standard oral lipid supplement consisting of MCT-oil.

### Study population

Inclusion criteria:

- Infants born at Oslo University Hospital with GA < 29 weeks
- Less than 48 h of age at inclusion
- Signed informed consent documented according to ICH GCP, and national/local regulations

Exclusion criteria

- Major congenital malformations
- Chromosomal abnormalities and other genetic diseases diagnosed prenatally or detected during the study period
- Critical illness with short life expectancy

### Intervention

**Fig. 1:**
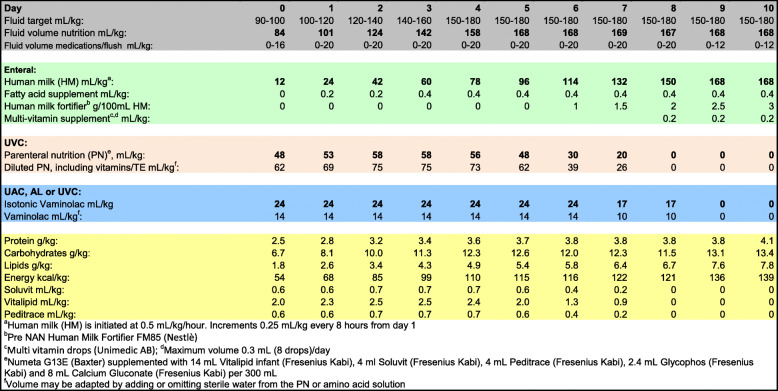
Nutritional protocol

The nutritional management of the study population is standardized. Initially, the infants receive a combination of PN and human milk, with a gradual increase of nutrients in line with international recommendations (Fig. 1). For this study the nutritional supplements Formulaid™ and MCT-oil™ are defined as investigational nutritional products (INPs). Formulaid™ is an oil-based enteral supplement, derived from fungi and microalgae, containing ARA and DHA in a 2:1 ratio. MCT-oil™ (Nutricia) contains medium chain triglycerides, based on coconut- and palm oil, and is currently the standard lipid supplement in our department to enhance enteral energy supply. The increments of human milk are 12–24 mL/kg/day in both study groups. Full enteral nutrition is defined as 170 mL/kg/day of human milk fortified with PreNAN FM 85® (Nestlé) 3 g/100 mL. The standard intravenous solution used for PN in extremely preterm infants in our department is Numeta G13 E™ (Baxter). Numeta G13E contains a soybean and olive oil emulsion very low in ARA and DHA. To improve the intake of these fatty acids in the intervention group, half-strength enteral supplementation with ARA and DHA is started on the second day of life, and advanced to target, i.e. 0,4 ml/kg (ARA 100 and DHA 50 mg/kg/d), from day 4 and onwards. The infants in the control group receive 0.2 ml/kg of MCT-oil with an increase to 0.4 ml/kg on day 4. It is the responsibility of the physicians of the trial to prescribe the study supplements in the infant’s medication chart. The dose is adjusted weekly against the current weight of the infant; birthweight is used until birthweight is regained. The enteral FA supplementation is administrated in the feeding tube as a daily bolus until 36 weeks PMA. If enteral feeds are stopped by any reason, the FA supplement is also withheld, but administration is recommenced as soon as enteral feeds are reestablished. To ensure compliance, the pharmacist allocated to the trial monitors medication charts and number of doses given.

### Randomization

For allocation of the participants to the two treatment groups, a computer-generated list of random numbers is used. The randomization is restricted by blocking and stratification. The block size is determined by the statistician (in the range of 4 to 10) and the information regarding the block size is kept in a separate document unavailable to those who enroll patients or assign treatment. To decrease the risk of unbalanced baseline characteristics, we stratify the randomization by growth status at birth (small for gestational age or not). Within each block, the allocation ratio is 1:1. After informed consent has been obtained, the attending physician or local investigator contacts the pharmacy for allocation consignment. In case of multiple births, the infants are allocated to same treatment by randomizing only the first infant.

### Subject withdrawal

All patients randomized are included in the study population. Patients who withdraw, or are withdrawn from the study after randomization, cannot be replaced. Patients withdrawn from the study stop further treatment, but already obtained data maintain in the study and will be analysed in line with the principles of intention-to-treat (Consort guidelines [65]). Patients may be withdrawn by the attending physician or the principal investigator for safety reasons, major protocol deviations or deteriorations in the patient’s condition which warrants study medication discontinuation. These infants remain in the study and their outcomes will be included in the intention-to treat analysis. The reason for patient discontinuation is recorded and the investigator is obliged to follow up any significant adverse event.

### Blinding

All investigators, staff, and participants, except for the pharmacists, are kept blind to fatty acid assignment of the participants. The enteral supplements are matched in volume. Since the colors of the lipid emulsions are not matched, we use amber syringes and cover the feeding tube when the emulsion is given to the infants.

### Primary outcome

The primary outcome is brain maturation at TEA, assessed by diffusion MRI using Tract-Based Spatial Statistics (TBSS). The diffusion MRI (MD) protocol consists of a multi-b-shell sequence allowing for higher order diffusion imaging as well as tractography and structural connectivity evaluation. The MRI protocol additionally includes multi-echo MP2RAGE sequences for surface-based and volumetric analyses as well as T1-relaxometry, which are also associated to maturation [66, 67]. 3D-T2 weighted images, allows for assessment of pathologies and the morbidity score ad modum Kidokoro [58].

### Secondary outcomes

**Fig. 2:**
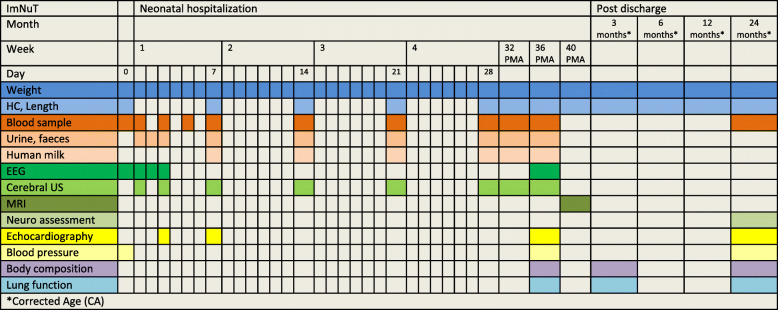
Summary of study assessments and procedures

1. Neurodevelopment assessed by electroencephalogram (EEG), cerebral ultrasound and neuropsychological testing. We monitor brain activity by continuous EEG during the first 3 days of life and at 36 weeks PMA. We will look at EEG maturational changes and assess background activity (total absolute band power) and connectivity (directed transfer function). Cerebral ultrasound (C-US) describing parenchymal pathology and cerebral blood flow are done according to the routines in our department. At 2 years corrected age (CA) we will perform a standardized neurodevelopmental evaluation including cognitive, behavioral and motor development, as well as a full neurological examination.
2. Quality of growth: Weight nadir, time to regain birth weight, change in HC, weight, length (z-scores), growth velocity from birth to 28 days and 36 weeks PMA. Anthropometric assessments at term age, 3, 6, 12 and 24 months CA. Body composition (fat and fat-free mass) assessed at 36 weeks PMA and 3 months CA by using PEA POD, a non-invasive Air Displacement Plethysmography system. Infant PEA POD measurements will be followed up with body composition measurements (Seca mBCA 525) at 2 years CA.
3. The cumulative incidence of neonatal morbidities associated with inflammation: BPD (defined as oxygen supplementation or respiratory support at 36 weeks PMA), PDA, ROP, NEC, IVH, WMI, and late-onset septicemia (LOS). Neonatal morbidities are registered in our database and graded according to standard classifications [68–73]. Severe ROP is defined as grade 3 or more. Culture-negative LOS is defined as presence of clinical symptoms plus abnormal white blood cell count, CRP ≥30 or antibiotic treatment for ≥5 days.
4. The frequency and severity of other neonatal morbidities, including postnatal growth restriction and hyperglycemia.
5. Cardiovascular health assessed by Doppler echocardiography and blood pressure measurement. Myocardial function is examined day 3 and 7, at 36 weeks PMA and 2 years CA by the use of two-dimensional echocardiography, including speckle tracking (2D strain) and tissue Doppler imaging. We will compare measurements against healthy term infants examined at the same time-points (REK-nr.2018/393). Blood pressure is measured at birth, at 36 weeks PMA and at 2 years CA.
6. Lung function assessed by tidal-flow-volume measurement at 36 weeks PMA, 3 months and 2 years CA. We also register the need for supplemental oxygen, number of days on mechanical ventilation or non-invasive respiratory support, and cumulative dose of postnatal steroids.
7. Inflammatory, metabolic and nutrient markers in blood, saliva, urine, feces and human milk.
  We register the results of routine blood samples during hospital stay. Markers of nutritional status, including vitamin A and D, are assessed at 28 days and 36 weeks PMA. Dried blood spots samples are collected daily during the first 5 days of life and then weekly for analyses of FAs, markers of inflammation i.e. interleukin-1, − 6 -10 and TNF-alpha, amino acids (branched chain amino acids (BCAA), glutamine, glutamate, arginine, asparagine, aspartate, phenylalanine, hydroxyproline, taurine, ornithine and citrullinec [74]), acyl carnitines and stress hormones. Both targeted and untargeted metabolic analyses for identification of individual compounds, ratios and affected biochemical pathways will be performed to investigate and describe metabolic differences based on the study interventions, but also to unravel potential wider differences between subgroups of infants. During hospital stay samples of urine and faeces are collected regularly. The urine samples will be used with the blood samples to evaluate metabolic and electrolyte changes during the first weeks of life. Faecal samples will be used to study the early faecal microbiota development. We collect tracheal samples of the intubated infants and nasopharyngeal samples in infants receiving non-invasive respiratory support. The samples will be included in the inflammatory and stress analyses. Small amounts of human milk are collected for analyses of nutrients.
8. Accommodation of the recent updated nutrient recommendations. Major efforts are made to ensure effective implementation of the nutritional protocol. The intake of all enteral and nutrients including fluid supply, carrier solutions and blood products are registered prospectively until 36 weeks PMA and the infants are monitored closely for electrolyte and mineral disturbances so that necessary adjustments can be done. We use the nutrition database Nutrium® (www.nutrium.se, Nutrium AB, Umeå, Sweden) for the nutritional calculations.
  Study assessments and procedures are summarized in Fig. 2.

### Sample size

We have used data from the PRENU-cohort for our determination of sample size. The PRENU study showed decreased regional white matter mean diffusivity (MD), suggestive of improved maturation of cerebral connective tracts in the intervention group [15]. The MD in the superior longitudinal fasciculi was 1.18 × 10–3 (6.18 × 10–2) in the intervention group and 1.25 × 10–3 (5.70 × 10–2) in the control group. We consider a 0.04 difference in mean MD as clinically significant. With a power of 80% and a significance level of 5%, 36 infants are required in each group (estimated sample size for two-sample comparison of means). Assuming 40% loss to follow-up (20% mortality/withdrawals/unavailability for scans, 20% non-interpretable images), 120 infants need to be included.

### Data management

Registration of patient data is carried out in accordance with national personal data laws. The Clinical Data Management System (CDMS) used for the eCRF in this study is VieDoc. Data is stored in a de-identified manner where each study participant is recognisable by a unique trial subject number. The investigator is responsible for the secure retention of the patient identification and the code list. All information concerning the study is inaccessible to unauthorized personnel and the data management procedures are performed in accordance with the ICH guidelines. Data management personnel perform both manual eCRF review and review of additional electronic edit checks to ensure that the data are complete, consistent and reasonable. Any changes to signed eCRFs must be approved and resigned by the Investigator. The data will be stored until 15 years after the last patient has completed last visit. Participant confidentiality is strictly held in trust by the responsible investigators and research staff.

### Statistical analysis

The primary outcome, TBSS analysis of MD in major white matter tracts at TEA, will be performed by one investigator without knowledge of group affiliation. Other MRI based outcomes will be analysed using voxel-based or “region of interest” (ROI) based analyses and connectivity maturation will be assessed using both global and regional network metrics comparing the two groups [75]. To evaluate the differences between groups on secondary outcomes we will use Student’s t test for continuous variables and a χ2 test or Fisher’s exact test for categorical variables. The Mann-Whitney U test will be used for variables not normally distributed. We will also use multiple linear regression analysis to adjust for important covariates in our analyses and methods for repeated measures will be used as well as parametrical and non-parametrical methods where relevant. Nutrient intake will be compared between the two groups and investigated in relation to clinical outcomes, growth, immune- and metabolic response. A *p*-value of < 0.05 will be considered statistical significant. Multiple hypothesis testing will be performed with p-value correction.

### Data analysis

The first main statistical analysis is planned when the last included patient has completed neuroimaging (MRI) at term equivalent age. The final analyses will be predefined before we unblind the data.

### Adverse events and safety monitoring

The trial investigator is responsible for the detection and documentation of events meeting the criteria and definition of an adverse event (AE) or serious adverse event (SAE). Any SAE is reported immediately to the sponsor and principal investigator. The collection and reporting of AEs are in line with Good Clinical Practice (GCP) guidelines on reporting of “Adverse Reactions” in clinical trials. The randomization code for a participant may be unblinded in a case of emergency. Code breaking is only to be used in circumstances where knowledge about the allocated treatment group is necessary for appropriate treatment of a serious adverse reaction and the Principal Investigator must be contacted. An external Data Monitoring Committee (DMC) consisting of a senior neonatal consultant, a pediatrician and a statistician with experience with clinical trials are monitoring the safety and scientific soundness of the trial. To evaluate possible negative effects of the nutritional interventions, a pre-planned safety interim analysis was performed by the DMC in September 2019, after 50 infants had been included. The unanimous decision of the committee was that there are no safety concerns warranting the trial to be terminated and they recommended the study to continue as planned.

## Discussion

Improvements in neonatal care have led to rising survival rates of extremely premature born infants [76], however, the rate of severe medical disabilities increases significantly with decreasing GA [76–78] and preventive measures to reduce neurodevelopmental sequelae, postnatal growth failure and inflammatory mediated diseases are most wanted. ARA and DHA are considered essential during early development and studies suggest that supplementation with ARA and DHA has structural effects on brain growth and maturation [18, 20, 21] and reduce severity of BPD, ROP, NEC and WMI by affecting the immune response [30–32]. In this study, we randomize preterm infants before 29 weeks GA to receive an enteral supplement consisting of either ARA and DHA or MCT-oil during neonatal hospitalization. A double blind RCT is the best scientific method to evaluate the efficacy of a treatment and minimize confounders, and the results of our study will thus be important in defining ARA and DHA requirements and to guide recommendation for supplements to extremely premature infants. If supplementation with ARA and DHA reduces the incidence of major neonatal morbidities, this will have great impact for future premature infants and their caregivers, given that even small improvements in cognition and neurodevelopmental health improves the children’s possibilities in future life [79].

Historically there have been many clinical misadventures due to lack of clinical paediatric research [80]. Research in infants is important and particularly advocated if it provides information that will improve the understanding or treatment of a condition, or if the interventions studied involves diagnostic procedures [81]. Our study participants constitute an especially vulnerable group of patients, since extremely premature born infants often are exposed to critical illness and an uncertain prognosis. The need of scientific knowledge involving such high risk participants rise several ethical issues in balancing the benefits and burdens [82].

The study design was chosen to enable insight into the complex interaction between inflammatory, metabolic and nutritional factors and how early events affect growth, metabolic functions and overall development. Several of the MRI sequences used in the study have, to our knowledge, never been implemented in infants in Norway before. Likewise, there exists no good method to automatically segment and measure the brain regions in infants and the study will assist in developing such a method. Thus, it is our hope that future patients, other studies and clinicians will benefit from these new methods.

Optimal management of nutrition to premature born infants remains a challenge. Updated guidelines on pediatric parenteral nutrition were recently published [16]. By implementing a standardized nutrition protocol this study will contribute to evaluate safety and efficacy of current guidelines. The at hand registering of actual supplied nutrients for all parenteral and enteral sources probably promotes an effective implementation of the nutritional protocol and leads to reduced practice variation in prescription of PN within the department. Several studies have shown that implementation of standardized nutritional protocols in the intensive neonatal care unit improves growth outcomes and reduces the incidence of comorbidities such as NEC and sepsis [83, 84], and might thus in itself be of benefit for all participating infants.

The participation of extremely preterm infants relies on the parents’ ability to make a decision under stressful conditions. By screening the maternal ward we try to obtain informed consent before the onset of labour. We do not know if supplementation with ARA and DHA is superior to supplementation with MCT-oil, so the direct benefit for each participating infant is unknown. However, studies show generally that participants of research find it more beneficial than harmful [85]. Parents of preterm infants with a birth weight < 1500 g included in a previous randomized nutritional intervention trial conducted in our institution reported better quality of life while in the neonatal unit and less sleeping problems and more energy at 3.5 years post-trial compared to parents without trial participants [86].

We included our first study participant to the ImNuT trial in April 2018. Recruitment is on-going with the aim to include the total sample size of 120 infants by the end of 2020. The first main statistical analysis is planned in 2021. We plan to publish the results of this study in peer-reviewed journals and present data at national and international conferences. The results of this study will also be submitted to the Ethics Committee according to EU and national regulations. Designation of authorship will follow the Vancouver criteria (recommendations of the international Committee of Medical Journal Editors, ICMJE).

## Data Availability

Data sharing is not applicable to this article.

## Abbreviations

ARA: Arachidonic acid
BPD: Bronchopulmonary dysplasia
CA: Corrected age
DHA: Docosahexaenoic acid
EPA: Eicosapentaenoic acid
FA: Fatty acid
HC: Head circumference
IVH: Intraventricular hemorrhage
LC-PUFA: Long-chain polyunsaturated fatty acid
MCT: Medium chain triglycerides
MD: Mean diffusivity
MRI: Magnetic resonance imaging
NEC: Necrotizing enterocolitis
PDA: Patent ductus arteriosus
PMA: Postmenstrual age
PN: Parenteral nutrition
ROP: Retinopathy of prematurity
TEA: Term equivalent age
WMI: White matter injury
